# The Relationship between Turgor Pressure Change and Cell Hydraulics of Midrib Parenchyma Cells in the Leaves of *Zea mays*

**DOI:** 10.3390/cells7100180

**Published:** 2018-10-22

**Authors:** Yangmin X. Kim, Burkhard Stumpf, Jwakyung Sung, Sang Joon Lee

**Affiliations:** 1 Department of Plant Ecology, Bayreuth University, D-95440 Bayreuth, Germany; yangmink@korea.kr (Y.X.K.); burkhard.stumpf@uni-bayreuth.de (B.S.); 2 Division of Soil and Fertilizer, National Institute of Agricultural Sciences, RDA, Wanju 55365, Jeollabuk-do, Korea; 3 Department of Mechanical Engineering, Pohang University of Science and Technology (POSTECH), Pohang 37673, Gyeongbuk, Korea

**Keywords:** aquaporin, cell pressure probe, hydraulic conductivity, *Zea mays* L.

## Abstract

Leaf dehydration decreases water potential and cell turgor pressure. Therefore, changes in cell turgor pressure may regulate water transport across plant cell membranes. Using a cell pressure probe, the hydraulic properties of parenchyma cells in the midrib of maize (*Zea mays* L.) leaves were measured (half time $T_{1/2}^{}$ of water exchange in cells as a measure of hydraulic conductivity *Lp*). Using intact plants with root systems encased in a pressure chamber, the root systems were pressurized and the turgor pressure in leaf cells increased by increments up to 0.3 MPa. However, the increase in the cell turgor did not increase but stabilized $T_{1/2}^{}$ values. Increased water potential in leaf cells seemed to have stabilizing effects on the $T_{1/2}^{}$ probably due to enhanced water availability. When the cell turgor decreased by 0.1 MPa to 0.3 MPa with releasing the pressure in the pressure chamber, $T_{1/2}^{}$ was temporarily increased to a large degree, a factor of up to 13 within 30 min.

## 1. Introduction

Plant leaves experience various ranges of water potential in response to the changes in transpiration rate or soil water content. In response to such changes, plants need to adjust their ability to conduct water through leaves, the leaf hydraulic conductance [1]. The leaf hydraulic conductance (*K*_leaf_) is the inverse of the hydraulic resistance *R*_leaf_, and expressed in kg H_2_O s^−1^ MPa^−1^. Leaf dehydration has been known to decrease *K*_leaf_ [2,3,4]. One of the possible causes for the *K*_leaf_ decline with dehydration is the outside-xylem hydraulic conductance, which includes the cell hydraulic conductivity, *Lp* [5,6,7,8]. The cell *Lp* is a measure of how efficiently water is transported through a single cell normalized by cell area and expressed in m^−1^ s^−1^ MPa^−1^. However, the cause for the *K*_leaf_ decline has not been fully studied yet. The cell *Lp* could decline in response to the decrease of the cell turgor pressure by leaf dehydration [3,9]. Increasing evidence shows that the hydraulics in a single plant cell level are mainly regulated by water channels, aquaporins (AQPs) [10,11,12,13,14,15,16,16,18]. In response to biotic or/and abiotic stress, AQPs can either increase or decrease the cell *Lp* by either opening or closing (‘gating’) in a short-term response. On the other hand, in a long-term response, the *de novo* expression of AQPs can increase cell *Lp* or the development of the apoplastic barrier can decrease cell *Lp* [14,15,19,20,21]. 

Turgor pressure has been suspected to be a signal of gating AQPs [22,23]. A previous study showed that change in the turgor pressure or mechanical stimuli affected the cell *Lp* [24]. Moreover, the cell *Lp* change has been shown to be attributed to the action on AQPs [9,24,25,26]. Wan et al. [24] reported that both positive and negative pressure pulses decreased the cell *Lp* and that the action of AQPs was involved. They suggested a model in which the mechanical stimuli (pressure pulses) induced water flux and closed the AQPs. Kim and Steudle [9] investigated the change in the cell *Lp* in response to illumination, which reduced the turgor pressure because of the increase in leaf transpiration. They reported that the cell *Lp* was first increased by light and then decreased as the turgor pressure decreased. In this case, the light and turgor pressure changed together, so the effects caused by light and turgor coexisted and separation of the effects by light and turgor was difficult. When Kim and Steudle [9] maintained the turgor constant during illumination to eliminate the turgor effect, the change in light increased the cell *Lp*. This result indirectly demonstrated that the decrease in turgor pressure led to the decrease in the cell *Lp*. However, it was not a direct measurement of the effect of turgor pressure.

In the present study, we aimed to examine the effect of turgor pressure change on the water permeability of parenchyma cells in the midrib of a maize leaf using a cell pressure probe [27,28]. We investigated the effects of the cell turgor pressure on the water permeability of parenchyma cells in the midrib of a maize leaf. We varied the turgor pressure by applying pressure to the root system encased in a pressure chamber to manipulate the root pressure and the leaf cell turgor. To increase the cell turgor, the pressure chamber pressurized the root system, and then the pressure in the pressure chamber was released to the atmospheric pressure. After changing the pressure, cell *Lp* values were continuously measured. This measurement result showed the kinetics of cell *Lp* and allowed the discussion in terms of the gating of AQPs.

## 2. Materials and Methods

### 2.1. Plant Material

Corn (*Zea mays* L. cv. monitor) plants were grown in plastic pots with soil in a greenhouse of Bayreuth University, Germany as described in Kim and Steudle [9]. When plants were 4 to 8 weeks old, the cell pressure probe measurements were performed on parenchyma cells in the midrib region of the leaves, which were fourth or fifth leaves counting from the oldest. The cells were located 100–200 mm behind the leaf tip. Material used in this study was the same tissue of the plants of a similar age, as in Kim and Steudle [9].

### 2.2. Experimental Setup Using a Cell Pressure Probe

As described earlier [9], parenchyma cells in the midrib were punctured by a microcapillary of a cell pressure probe (CPP). The capillary with a fine tip of about 6 µm in diameter was filled with silicon oil (oil type AS4 from Wacker, Munich, Germany). The measurements of the cell turgor pressure (*P*) using CPP are described in Kim and Steudle [9]. Hydrostatic relaxation of turgor was performed and the half time of the hydrostatic relaxation, $T_{1/2}^{}$, was obtained to calculate the hydraulic conductivity of cell membranes (*Lp*) using the Equation (1) in Kim and Steudle [9]. The volumetric elastic modulus (*ε*) was calculated using the pressure change and relative volume change by Equation (2) in Kim and Steudle [9]. In the calculation of *Lp* and *ε*, we used average values of cell diameter and length from Kim and Steudle [9], who used the same type of cells located in a similar position from the plants of a similar age. In most of cases, $T_{1/2}^{}$ was used to indicate the change in *Lp* because *ε* did not change significantly during the whole measurements even though there is a change in turgor pressure (see Results). The half time $T_{1/2}^{}$ is inversely proportional to *Lp*, that is, large $T_{1/2}^{}$ means small *Lp*. During the hydrostatic relaxation, the size of the pressure peak was maintained at less than 0.1 MPa to avoid closing aquaporins by large pressure pulses, as reported in Wan et al. [24]. After puncturing cells, $T_{1/2}^{}$ varied in the leaf cells of intact corn plants grown in soil [9]. Less than half of the population of cells measured in this study had small $T_{1/2}^{}$ values of approximately 1 s after a transient increase in $T_{1/2}^{}$ caused by the cell puncture, as discussed later. For those cells having small $T_{1/2}^{}$ values, we checked whether or not $T_{1/2}^{}$ was affected by the change in turgor pressure. Further information on the CPP measurement is described in previous studies [29,30,31].

### 2.3. Pressurization Experiment

The root system of an intact corn plant was encased in a pressure chamber and light lamp (Siemens AG, Frankfurt, Germany) was installed above the plant to illuminate the whole plant. It was the same set-up used in Kim and Steudle [9]. The root system was pressurized with the increment of pressure in the range of 0.05 MPa–0.1 MPa (small), 0.11 MPa–0.2 MPa (medium), or 0.21 MPa–0.3 MPa (large). Pressurizing the root caused to increase cell turgor pressure in leaves (see Results). Hydrostatic relaxations ($T_{1/2}^{}$) were evaluated before and after the root system was pressurized. After the root was pressurized, $T_{1/2}^{}$ values of hydrostatic relaxations were evaluated only when the turgor value was rather constant to eliminate the errors in determining the $T_{1/2}^{}$ under changing turgor. When pressure was released to the atmospheric pressure, the cell turgor pressure returned to the original level, and then $T_{1/2}^{}$ was analyzed.

## 3. Results

### 3.1. Pressure Chamber Pressure vs. Cell Turgor Pressure

**Figure 1:**
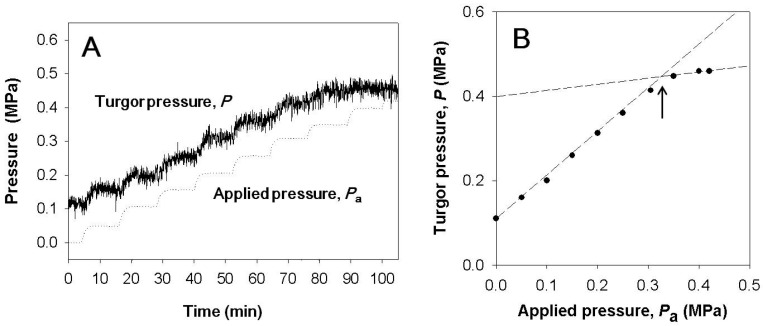
(**A**) Variation of the turgor pressure (*P*) with respect to the pressure applied to the root system (*P*_a_). Before time zero, the plant was illuminated with the light intensity of 160 μmol m^−2^ s^−1^ at the level of measured cell and the cell turgor was about 0.1 MPa, which was lower than thatwithout illumination. (**B**) Applied pressure (*P*_a_) vs. turgor pressure was plotted from values in (**A**). Before starting guttation, the increment of the turgor pressure was equal to that of the pressure applied to the pressure chamber. Once guttation occurred, the increment of the turgor was smaller than that of the applied pressure. The arrow indicates the point where guttation started.

**Figure 2:**
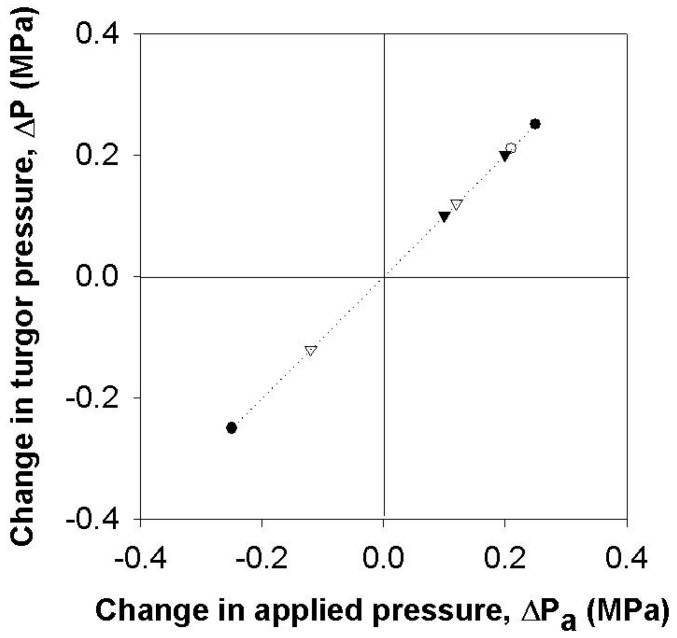
Change in the turgor pressure (Δ*P*) with respect to the changes in pressure applied to the root system (Δ*P*_a_). Different symbols indicate different cells and only cells with no guttation were included. The dotted line shows the 1:1 relationship in pressure changes.

For intact plants, the root pressure was modified to change leaf cell turgor. When a certain level of pressure was applied to the root system, the cell turgor pressure increased with the ratio of 1:1 (Figure 1 and Figure 2). Before time zero, the whole plant in Figure 1 was illuminated with the light intensity of 160 µmol m^−2^ s^−1^ at the level of the measured cell and the cell turgor was about 0.1 MPa, which was lower than it was without illumination. When the pressure applied to the root was bigger than 0.3 MPa, guttation was observed. In the occurrence of guttation, the responses in the turgor were no longer 1:1 (Figure 1).

**Figure 3:**
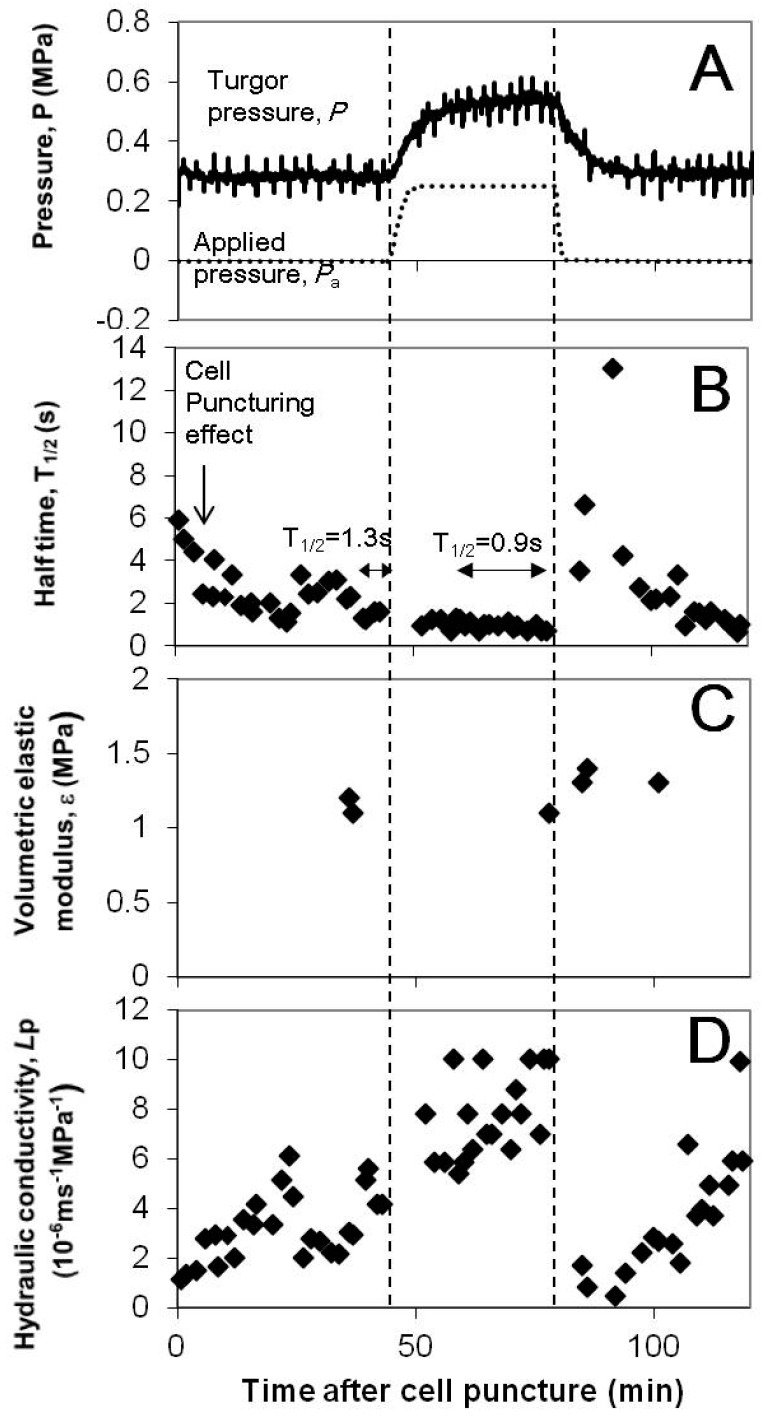
(**A**) Temporal variations of the leaf cell turgor (*P*; solid curve) and the pressure applied to the pressure chamber (*P*_a_; dashed curve). The atmospheric pressure is zero. Time zero is when a cell was punctured by a capillary of a cell pressure probe. The vertical lines indicate the duration of pressurization to the root system. The pressure in the pressure chamber was increased by the increment of 0.25 MPa at the first dashed line, and the cell turgor pressure was increased by the increment of 0.25 MPa (large range). When the chamber pressure started to be released to the atmospheric pressure at the second dashed line, the turgor pressure decreased and finally returned to the original value. (**B**) Effect of the change in the turgor pressure on the half time of hydrostatic relaxation, $T_{1/2}^{}$. Hydrostatic relaxations appear as pressure peaks in (**A**). In the beginning after puncturing the cell, $T_{1/2}^{}$ had large values and reduced to a small value around 1 s at 40 min due to puncturing effects (see text). The turgor pressure that increased by the increment of 0.25 MPa did not temporarily increase $T_{1/2}^{}$ for 35 min. However, the turgor pressure decreased by 0. 25 MPa caused a transient increase in $T_{1/2}^{}$. Nevertheless, the half time $T_{1/2}^{}$ finally recovered to a small value within 30 min. (**C**) Effect of the change in the turgor pressure on the volumetric elastic modulus, *ε*. The elastic modulus did not change much, even though turgor pressure changed. This justifies using $T_{1/2}^{}$ as a direct measure of change in *Lp*. (**D**) The hydraulic conductivity, *Lp*, was calculated from the $T_{1/2}^{}$ and *ε* in (**B**,**C**), assuming the cell is a cylinder with a diameter of 63 μm and a length of 76 μm as in Kim and Steudle [9].

A representative curve of turgor variation upon pressurizing the root chamber is shown in Figure 3A. When a pressure of 0.25 MPa was applied to the pressure chamber, the turgor pressure increased by the same amount (0.25MPa). The changes in turgor pressure were much slower than those of pressure in the root pressure chamber, that is, half time of the pressure change in increasing pressure: 80 s and 220 s for the root chamber pressure and turgor pressure, respectively.

### 3.2. Cell Hydraulic Conductivity, Lp and the Half Time of Hydrostatic Relaxation, $T_{1/2}^{}$

The *Lp* calculation needed $T_{1/2}^{}$ of hydrostatic relaxation, the volumetric elastic modulus (*ε*), and cell geometry. To calculate *Lp* for each hydrostatic relaxation in Figure 3A, $T_{1/2}^{}$ of the relaxation curve was used and *ε* value at each turgor pressure was used (Figure 3B,C). For cell geometry, it was assumed that the cell is a cylinder with a diameter of 63 μm and a length of 76 μm as in Kim and Steudle [9]. The elastic modulus did not change much along the change in turgor pressure (Figure 3C; *ε* = 1.1–1.4 MPa). Therefore, using $T_{1/2}^{}$ as a direct measure of change in *Lp* is justified and we investigated changes in $T_{1/2}^{}$ upon turgor pressure alterations.

### 3.3. Effect of Turgor Pressure Alterations on $T_{1/2}^{}$

Turgor pressure was increased by the increment of 0.05 to 0.1 MPa (small), 0.11 to 0.2 MPa (medium), or 0.21 to 0.3 MPa (large), and then it was decreased back to the original value. A representative case in Figure 3B shows that $T_{1/2}^{}$ initially varied after puncturing of a cell and reduced to a small value around 1 s at 40 min. The increase in turgor pressure of the cell by the increment of 0.25 MPa (large range) stabilized $T_{1/2}^{}$. However, when the turgor of the cell decreased by 0.25 MPa and returned to the original turgor pressure in the beginning of the measurement, there was an increase in $T_{1/2}^{}$ by a factor of bigger than 10. The $T_{1/2}^{}$ s were finally recovered to small values within 30 min. The transient increase in $T_{1/2}^{}$ by the large turgor decrease had some analogy to the effect of pressure pulses [24].

### 3.4. Effect of Turgor Pressure Increase on $T_{1/2}^{}$

**Figure 4:**
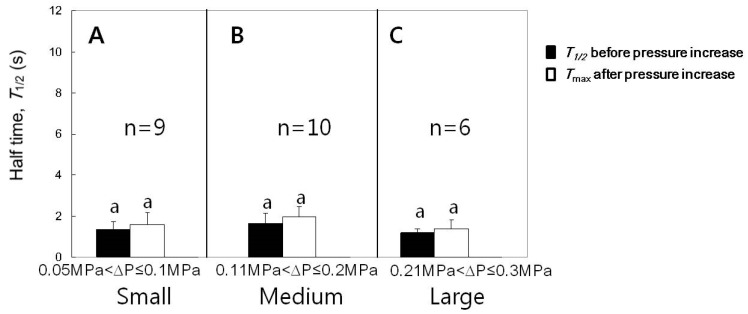
Effects of the increased cell turgor pressure on the maximum $T_{1/2}^{}$ ($T_{\max}^{}$) for cells with $T_{1/2}^{}$ of approximately 1 s. (**A**–**C**) The increases of the turgor by the increments in small, medium, or large ranges did not increase $T_{1/2}^{}$. Mean values ± SD were plotted. The significance in differences was tested and noted with different alphabets (n = 6–10 cells, paired *t*-tests for each range of turgor pressure increase, *p* < 0.05).

**Figure 5:**
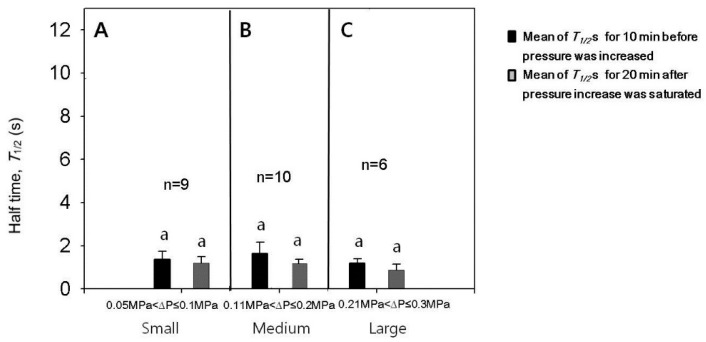
Comparison of the mean values of $T_{1/2}^{}$ for 20 min after the saturation of turgor increase with those for 10 min before the turgor increase ($T_{1/2}^{}$ recovered from a puncturing effect; (see text) for cells with $T_{1/2}^{}$ of approximately 1 s using 6 to 10 cells. The increases of the turgor by the increments in small (**A**), medium (**B**) or large (**C**) ranges tended to decrease the mean values of $T_{1/2}^{}$, which are indicated with double-headed arrows in Figure 3B. However, the decrease was not so statistically significant for repeated cells. Mean values ± SD were plotted. The significance in differences was tested and noted with different alphabets (n = 6–10 cells, paired *t*-tests for each range of turgor pressure increase, *p* < 0.05).

The experiments for the case of turgor pressure increase were conducted with varying increments in pressure increase and the results are summarized in Figure 4 and Figure 5. Single cells were employed to read $T_{1/2}^{}$ values before and after increasing the turgor pressure. After the cell puncture, $T_{1/2}^{}$ data were varied in the leaf cells of intact corn plants in a stochastic manner [9]. Less than half of the population of cells measured in this study had small $T_{1/2}^{}$ values of approximately 1 s after a transient increase in $T_{1/2}^{}$ caused by the cell puncture, as illustrated in Figure 3B. For those cells having small $T_{1/2}^{}$ values after the lapse of some time, we checked whether or not $T_{1/2}^{}$ values were affected by the change in turgor pressure. The increments of turgor pressure increase were classified into three ranges; small (0.05 to 0.1 MPa), medium (0.11 to 0.2 MPa), and large (0.21 to 0.3 MPa). We compared the $T_{1/2}^{}$ value just before the turgor pressure increased and the maximum $T_{1/2}^{}$ after the turgor increased to a stable value (*T*_max_). The increases in turgor pressure by small, medium, or large increments did not cause a temporary increase in the $T_{1/2}^{}$ (Figure 4; n = 6–10 cells, paired *t*-tests for each range of turgor pressure increase, *p* < 0.05). Aside from the temporary effect, the increases in turgor pressure by small, medium, or large increments tended to decrease the $T_{1/2}^{}$, however, the decreases were not so statistically significant (Figure 5; n = 6–10 cells, paired *t*-tests for each range of turgor increment, *p* < 0.05). For statistical analysis, we compared the mean values of $T_{1/2}^{}$ s for 10 min before the turgor increased and those for 20 min after the turgor pressure increased to a stable saturated value. The half times might further decrease in response to a longer period of high turgor pressure (see Section 4).

### 3.5. Effect of Decreases in Turgor Pressure Back to the Original Value on $T_{1/2}^{}$

**Figure 6:**
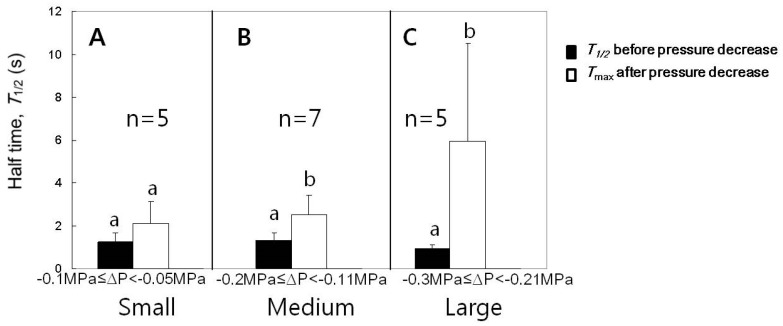
Effects of the decreased cell turgor pressure on the maximum $T_{1/2}^{}$ ($T_{\max}^{}$) for cells with $T_{1/2}^{}$ of approximately 1 s. (**A**) The decreases in the turgor by small ranges did not affect $T_{1/2}^{}$. (**B**) The decreases in the turgor by medium ranges increased $T_{1/2}^{}$. (**C**) The decreases in the turgor pressure by large ranges increased $T_{1/2}^{}$ in a bigger fold than in (**B**). Mean values ± SD were plotted. The significance in differences was tested and noted with different alphabets (n = 5–7 cells, paired *t*-tests for each range of turgor pressure decrease, *p* < 0.05).

The experiments for the case of turgor pressure decrease as seen in Figure 3 were conducted for varying pressure decreases and summarized in Figure 6. Following a single cell, we compared the $T_{1/2}^{}$ just before the turgor decreased and the maximum $T_{1/2}^{}$ after the turgor decreased to a stable value (*T*_max_). In response to the cell turgor decreases by small ranges (0.05 to 0.1 MPa), the changes in $T_{1/2}^{}$ were not significant. The decreases in the turgor pressure by medium (0.11 to 0.2 MPa) or large (0.21 to 0.3 MPa) ranges induced a transient increase in $T_{1/2}^{}$, which finally returned to the original value before decreasing the cell turgor pressure. The significances in the difference between $T_{1/2}^{}$ just before turgor decreased and *T*_max_ are shown in Figure 6 (n = 5–7 cells, paired t-tests for each range of turgor pressure decrease, *p* < 0.05).

## 4. Discussion

We observed that the pressurized root system increased the leaf cell turgor pressure and it rather stabilized leaf cell hydraulic conductivity (*Lp*). However, the return of the turgor to its original value by releasing the chamber pressure to the atmospheric pressure, except for the case of small changes up to 0.1 MPa, decreased the cell *Lp* temporarily. This transient pattern in cell *Lp* by pressure decrease was similar to what happened when pressure pulses were imposed on cortical cells of young corn roots in the study of Wan et al. [24]. These authors observed that root cell *Lp* decreased along both directions of the pressure pulses and they explained the phenomena by energy injection model. If the energy injection model was the only mechanism being applied here, this type of effect should have been observed after the turgor increased. However, it did not occur. Therefore, another type of co-working mechanism may play a role when the turgor pressure increases (water availability increases). Although the mechanism for the response of the cell *Lp* to the cell turgor pressure is still unknown, this study contributes to the revelation that the cell *Lp* responds to the changes in cell turgor.

**Figure 7:**
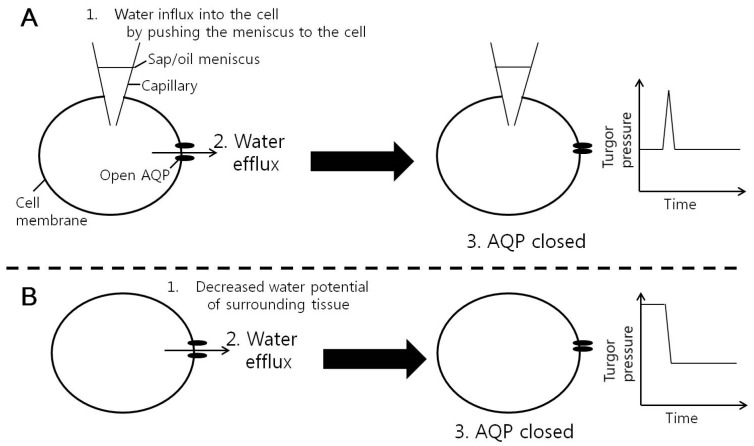
Energy injection model of the gating of aquaporins (AQPs) in membranes of corn cells by water flow passing the AQPs. (**A**) Pressure pulse effect in Wan et al. [24]. Moving the position of meniscus between sap and oil in a micro capillary of a cell pressure probe could produce a big pressure pulse and water flow through the AQPs. The water flow might inject energy to the AQP and cause a reversible mechanical deformation of the protein resulting in the closure of the channel. (**B**) In the present study, when water flow was produced by reducing the water potential in the surrounding leaf tissue by decreasing the root pressure, the water flow through the AQPs might close AQPs in the same way.

The next question concerns what the signal is to change the cell *Lp* when the cell turgor changes. The first candidate, which is most likely to affect the cell *Lp*, is the change in turgor pressure, which causes water flux and a mechanical stress. The decrease of the cell turgor pressure to its original value by releasing the root pressure was found to induce a temporary decrease in the cell *Lp.* This effect is analogous to that of Wan et al. [24] in which pressure pulses were imposed on the cortical cells of young corn roots. They observed a similar pattern of temporary decrease in cell *Lp*, which was more pronounced in larger pulses. They demonstrated this effect to be related to the aquaporins by using a heavy metal known to close the AQPs. According to the energy injection model of Wan et al. [24], the change in the turgor gives rise to a substantial water flux across the AQPs, transferring energy to the AQPs and closes them (Figure 7). There could be also another way of AQP gating in response to turgor pressure change. Johannson et al. [22] proposed that the Ca^2+^ channel could be mechanosensitively gated by changes in the turgor pressure. Cytoplasmic Ca^2+^ concentration has been known to be responsible for the gating of AQP by phosphorylation [22,32,33]. The next candidate is the water availability. If the pressure change was only detected, we could have observed the same effect caused by the increase in the cell turgor pressure. However, we did not observe it. The increase of turgor pressure rather stabilized the cell *Lp*, whose values fluctuated after the puncturing of the cell by a micro capillary of the cell pressure probe. In addition, there was a tendency of increasing cell *Lp* within 30 min after initiation of changing pressure, despite the negligible statistical differences. Therefore, another mechanism probably coexists to override the closure of AQPs in response to the increase in the water availability by increase in cell turgor pressure. The apoplastic water content near a cell may affect the local Ca^2+^ concentration or pH, and this may influence the gating of AQPs [22,32,33,34]. In a long-term response to high turgor pressure, the expression of AQPs might be involved and the cell *Lp* could respond differently [19].

At the level of leaf tissues, a similar trend of the decrease in *K*_leaf_ by the turgor pressure decrease was observed [23]. The authors considered the turgor pressure as a signal to decrease *K*_leaf_. They suspected the decrease in *K*_leaf_ was caused by the decrease in the cell *Lp*, by the closure of the AQPs in the bundle sheath. This speculation is in accordance with the result of the present study that the decrease in cell turgor decreases the parenchyma cell *Lp*, although there could be differences among different cell types. Moreover, *K*_leaf_ declines with dehydration, and the potential sources of *K*_leaf_ decline are known as xylem cavitation, xylem collapse, and the decline in conductance of extra-xylem paths [1,4]. Among these factors, recently the changes in extra-xylem paths such as AQPs in a bundle sheath were considered to play an important role to explain the *K*_leaf_ decline [5,6,7,8]. Especially for mild dehydration in which cells are still turgid, cell hydraulics would still have an important role in leaf tissue hydraulics. To investigate cell hydraulics, the cell pressure probe is a powerful tool to measure the activity of AQPs in a single living cell of intact plants, which encounter water deficit. However, the use of the cell pressure probe on cells having low turgor caused by water deficit is technically difficult. Either invention of a new technique or clever designs of the experiment, or both, are required to examine cell hydraulics in response to water deficit.

The transiency in the decrease of cell *Lp* by turgor decrease in the present experiment may sound inconsistent that dehydration decreases *K*_leaf_. However, the current experiment is not real leaf dehydration. It is a simulation of a turgor pressure decrease, which was first increased, to test the effect of water flux produced by pressure change. The increasing of a turgor implied that there could be an effect by the absolute value of turgor pressure, that is, increasing the turgor stabilized $T_{1/2}^{}$. If the turgor decreased compared to the original value, the effect could be different, that is, an effect of water flux and an effect of the decrease in the absolute value of turgor (water availability). This was observed by Kim and Steudle [9] by increasing the light intensity, and $T_{1/2}^{}$ initially decreased and then increased, but this was a co-effect of both light and turgor decrease. Other experiments reducing cell turgor, for example dehydration or application of osmoticum simulating water deficit, would give direct effects of the pressure decrease into a value lower than the original value.

The pattern of changes in cell turgor pressure by increasing the root pressure was similar to Wei et al. [28], who measured the responses in the xylem pressure using a pressure probe to the pressure applied to the root systems of corn plants. Cell pressure did not increase after guttation occurred as the xylem pressure did not increase after guttation occurred. The increased xylem pressure was delivered to the leaf cells. The increasing pressure in the leaf cells was slower than that in the xylem. This slowness is closely related to the distance from the cells to the xylem vessel and the cell hydraulic conductivity, *Lp* [27].

## 5. Conclusions

In conclusion, this study shows for the first time that the cell *Lp* temporarily decreased with the decrease in the cell turgor pressure. This study did not aim to identify the mechanism of *Lp* response to changes in turgor pressure. We postulated the involvement of gating of AQPs, because the cell *Lp* variation pattern of parenchyma cells in the midrib of maize leaves in response to the decreased cell turgor pressure was similar to that of cortical cells in young maize roots in response to pressure pulses in a previous study of Wan et al. [24]. They demonstrated the gating of AQPs by pressure pulses and heavy metals. However, we did not see the temporary increase in leaf cell *Lp* by increase of turgor pressure on the opposite of the case of turgor pressure decrease. These results support that both the changes in cell turgor pressure related to mechanical stimuli and the water availability may coordinate the hydraulic features of leaf cells in response to pressure changes.
